# Role of Muscarinic Receptor Signaling Processes in Specific Aspects of Morphine-Induced Respiratory Depression in Rats

**DOI:** 10.3390/ph19071101

**Published:** 2026-07-17

**Authors:** Paulina M. Getsy, Walter J. May, Santhosh M. Baby, Gregory A. Coffee, Yunguang Qiu, Feixiong Cheng, James N. Bates, Stephen J. Lewis

**Affiliations:** 1 Department of Pediatrics, Division of Pulmonology, Allergy and Immunology, School of Medicine, Biomedical Research Building, Case Western Reserve University, Cleveland, OH 44106, USA; pxg55@case.edu (P.M.G.); gac43@case.edu (G.A.C.); 2 Department of Pediatrics, University of Virginia, Charlottesville, VA 22903, USA; walterjmay@gmail.com; 3 Galleon Pharmaceuticals, Inc., 213 Witmer Road, Horsham, PA 19044, USA; babysanthosh@gmail.com; 4 Genomic Medicine Institute, Lerner Research Institute, Cleveland Clinic, Cleveland, OH 44195, USA; qiuy3@ccf.org (Y.Q.); chengf@ccf.org (F.C.); 5 Department of Molecular Medicine, Cleveland Clinic Lerner College of Medicine, Case Western Reserve University, Cleveland, OH 44195, USA; 6 Case Comprehensive Cancer Center, Case Western Reserve University School of Medicine, Cleveland, OH 44106, USA; 7 Department of Anesthesiology, University of Iowa Hospitals and Clinics, Iowa, IA 52242, USA; jnbates25@gmail.com; 8 Department of Pharmacology, Case Western Reserve University, Cleveland, OH 44106, USA

**Keywords:** morphine, atropine, muscarinic receptors, hypoxic-hypercapnic gas challenge, ventilatory parameters, rats

## Abstract

**Background/Objectives:** We examined the role of muscarinic cholinergic receptors in the ventilatory depressant effects of morphine in male Sprague Dawley rats, and in responses elicited by a hypoxic-hypercapnic gas (HH) challenge and upon return to room air. **Methods:** Ventilatory parameters were measured in freely moving rats by whole body plethysmography that continuously recorded these parameters. **Results:** The injection of morphine (10 mg/kg, IV) elicited a relatively pronounced depression of breathing, including sustained decreases in tidal volume, minute ventilation, peak inspiratory flow, and inspiratory and expiratory drives that were associated with marked increases in end inspiratory pause (EIP), expiratory flow at 50% expired tidal volume (EF_50_), and the rate of achieving peak expiratory flow (Rpef). Subsequent injection of the muscarinic receptor antagonist, atropine (1 mg/kg, IV), in morphine-treated rats did not affect the frequency of breathing, tidal volume, minute ventilation, inspiratory and expiratory times, end expiratory pause, peak inspiratory and expiratory flows, relaxation time, expiratory delay, apneic pause, inspiratory and expiratory drives, and the non-eupneic breathing index (*p* > 0.05 for all comparisons). In contrast, atropine dramatically reduced the morphine-induced increases in EIP, Rpef and EF_50_ (*p* < 0.05, for all comparisons). In addition, the changes in many ventilatory parameters (e.g., frequency of breathing, tidal volume and minute ventilation) that occurred during a subsequent HH-challenge and upon the return to room air were markedly reduced in atropine-treated rats (*p* < 0.05, for all comparisons). **Conclusions:** These findings suggest that muscarinic receptors play a limited, but important role, in the expression of the ventilatory effects of morphine in male rats and a substantial role in the expression of responses elicited during and following HH-challenge.

## 1. Introduction

The adverse effects of opioids on breathing and alveolar gas exchange complicates their clinical use [1,2,3,4]. Mechanisms responsible for opioid-induced respiratory depression (OIRD) are multifactorial and, as seen in Supplemental Table S1, many therapeutic modalities have been tried to elicit effective reversal of OIRD. The process underlying OIRD and/or the reversal of this phenomenon appear to involve alterations in the activities of (a) glutamatergic receptor-linked signaling cascades including NMDA receptors [5] and AMPA receptors [6,7], (b) 5-HT_1A_-, 5-HT_1A_-/5-HT_7_- and 5-HT_4_-receptor signaling cascades [8,9], (c) α_2_-adrenoceptor-initiated cell-signaling events [10] and (d) dopamine D1 receptor agonists [11,12,13]. Roles of plasma-membrane ion-channels and intracellular signaling molecules pertain to (a) K^+^-channels [14], such as large conductance Ca^2+^-activated K^+^ (BK) channels [15,16,17], KCNQ voltage-gated K^+^-channels [18] and G protein-gated inwardly rectifying K^+^ channels [19], (b) adenylate cyclases [20], (c) protein kinase A [11], and (d) phosphodiesterase [20,21]. Other entities include microglial inhibitors [22], glycyl-glutamine [23], thyrotropin releasing hormone and analogs [24], nitric oxide synthase [25], S-nitrosothiols [26] and, as summarized in Supplemental Table S1, thiols and L- and D-thiolesters [27,28].

Cholinergic mechanisms are involved in the expression of OIRD with acetylcholinesterase inhibitors [29,30,31] and nicotinic receptor agonists [32,33] showing efficacy in relieving some of the adverse effects of opioids. Cholinergic neurotransmission and cholinergic receptors (nicotinic and muscarinic) within the brain [34,35,36,37,38] and carotid bodies [39,40,41,42] have complex roles in the regulation of breathing and processing of chemoreceptor input. To date, potential roles of central or peripheral muscarinic receptors in the expression of OIRD in freely moving rats have not been explored. Our companion manuscript provides evidence that the ability of L-cysteine ethyl ester (L-CYSee) to overcome ventilatory depression to morphine is blunted by the co-administration of atropine [43], which is a peripherally/centrally acting antagonist of M1-M5 muscarinic receptors [44]. These M1-M5 receptors differ in their distribution and expression level in respiratory control centers in the brain [45,46,47,48] and carotid bodies [49,50,51,52,53] and the signal transduction processes they recruit. M1, M3 and M5 receptors are Gq/11 protein-linked and mainly located post-synaptically, and their activation stimulates phospholipase C-mediated increases in intracellular Ca^2+^ [45,48,52,53]. M2 and M4 receptors are Go/i protein-coupled receptors that are located pre- and post-synaptically, and upon activation they lower cellular concentrations of cAMP via inhibition of adenylyl cyclase [48,53].

The objective of the present study was to determine the role of muscarinic receptors in the ventilatory responses elicited by intravenous injection of morphine in unanesthetized adult male Sprague Dawley rats and in the subsequent ventilatory responses elicited by a hypoxic-hypercapnic (HH) challenge (rebreathing method), which are adversely impacted by morphine [54]. Two sets of rats were given morphine (10 mg/kg, IV) and after 15 min, one group received an injection of vehicle (saline) and the other a standard dose (1.0 mg/kg, IV) of atropine [55,56,57]. After 45 min, both groups of rats were subjected to a HH-challenge for 60 min followed by the return to room air for 30 min. Our findings demonstrate that muscarinic receptors play a limited role in the expression of the effects of morphine on ventilatory parameters in these rats whereas they have a more substantial role in the expression of the ventilatory responses elicited during and following HH-challenge.

## 2. Results

### 2.1. Baseline Ventilatory Values

The ages and body weights and baseline (pre) ventilatory parameters before injection of morphine in rats that then received an injection of vehicle (saline) or atropine are shown in Supplemental Table S3. There were no between-group differences for any parameter (*p* > 0.05, for all comparisons).

### 2.2. Ventilatory Studies

#### 2.2.1. Frequency of Breathing (Frequency), Tidal Volume (TV) and Minute Ventilation (MV)

**Figure 1:**
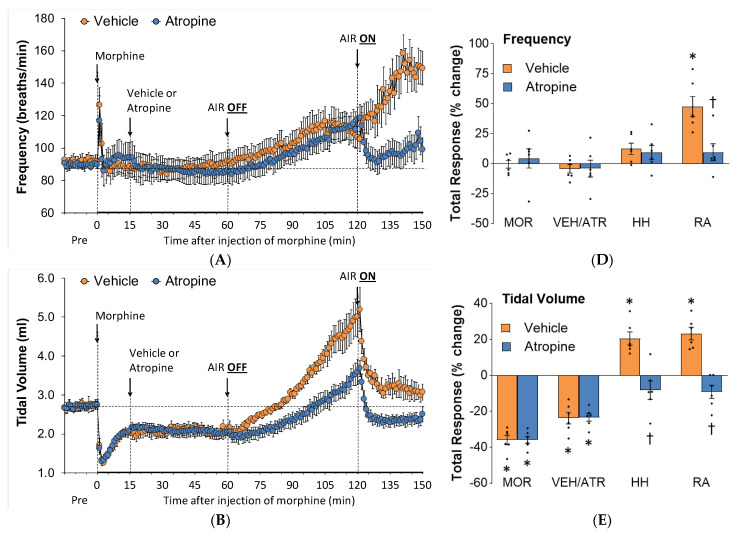

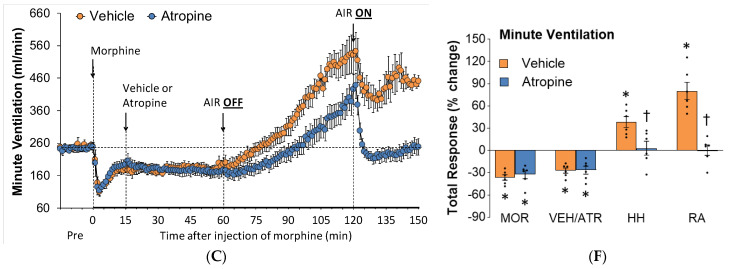
Values recorded for frequency of breathing (**A**), tidal volume (**B**), and minute ventilation (**C**) before (Pre), after injection of morphine (10 mg/kg, IV), and then subsequent injection of vehicle (VEH) or atropine (1 mg/kg, IV) followed by a hypoxic-hypercapnic gas challenge (AIR OFF) and return to room air (AIR ON). A summary of the total changes in frequency, tidal volume, and minute ventilation during each phase of the study are presented in (**D**–**F**), respectively (ATR, atropine; HH, hypoxic-hypercapnic gas challenge; RA, return to room air). The data are presented as mean ± SEM. There were 6 rats in each group. * *p* < 0.05, significant change from Pre-values. ^†^ *p* < 0.05, atropine versus vehicle.

As can be seen in Figure 1, the injection of morphine (10 mg/kg, IV) produced a transient increase in Frequency (panel A) but a substantial and sustained decrease in TV (Panel B) and therefore MV (Panel C). The subsequent injection of atropine (1 mg/kg, IV) did not affect these ventilatory actions of morphine. In contrast, the subsequent increases in Freq, TV and MV that occurred during the HH-challenge and upon return to room air were markedly diminished in atropine-treated rats compared to the vehicle-treated rats. Summaries of the total changes in Freq, TV and MV during each phase of the protocol (panels D–F, respectively) highlight the impact of atropine on the TV and MV responses that occurred during HH-challenge and upon return to room air.

#### 2.2.2. Inspiratory Time (Ti), Expiratory Time (Te) and Ti/Te

**Figure 2:**
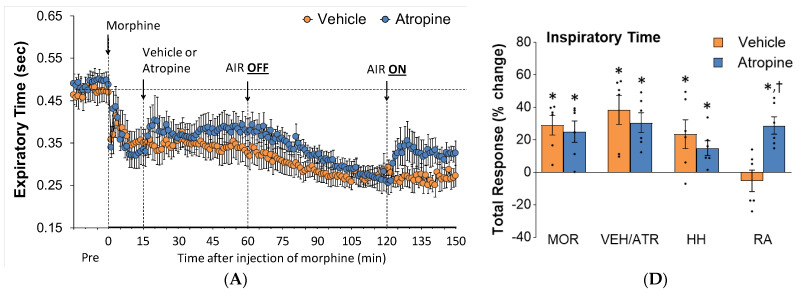

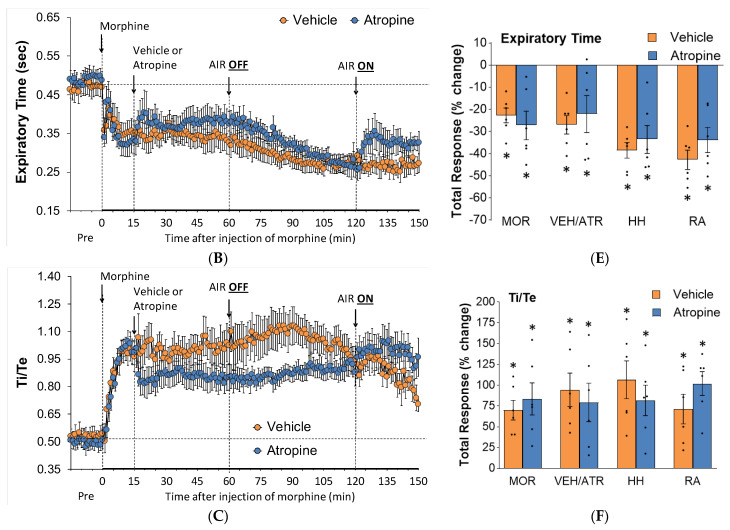
Values recorded for inspiratory time (**A**), expiratory time (**B**), and inspiratory time/expiratory time (Ti/Te) (**C**), before (Pre), after injection of morphine (10 mg/kg, IV), and then subsequent injection of vehicle (VEH) or atropine (1 mg/kg, IV) followed by a hypoxic-hypercapnic gas challenge (AIR OFF) and return to room air (AIR ON). A summary of the total changes in Ti, Te and Ti/Te during each phase of the study are presented in (**D**–**F**), respectively (ATR, atropine; HH, hypoxic-hypercapnic gas challenge; RA, return to room air). Data are presented as mean ± SEM. There were 6 rats in each group. * *p* < 0.05, significant change from Pre-values. ^†^ *p* < 0.05, atropine versus vehicle.

As seen in Figure 2, morphine elicited a transient decrease followed by a sustained increase in Ti (panel A), a transient decrease followed by a sustained decrease in Te (Panel B), and therefore a sustained increase in Ti/TE (Panel C). The subsequent injection of atropine minimally affected these parameters. The subsequent changes in Ti, Te and TI/Te that occurred during the HH-challenge and upon return to room air were similar in the atropine-treated and the vehicle-treated rats. Summaries of the total changes in Ti, Te and Ti/Te during each phase of the protocol (panels D–F, respectively) highlight the impact of atropine on the Ti responses that occurred upon return to room air.

#### 2.2.3. End Inspiratory Pause (EIP) and End Expiratory Pause (EEP)

**Figure 3:**
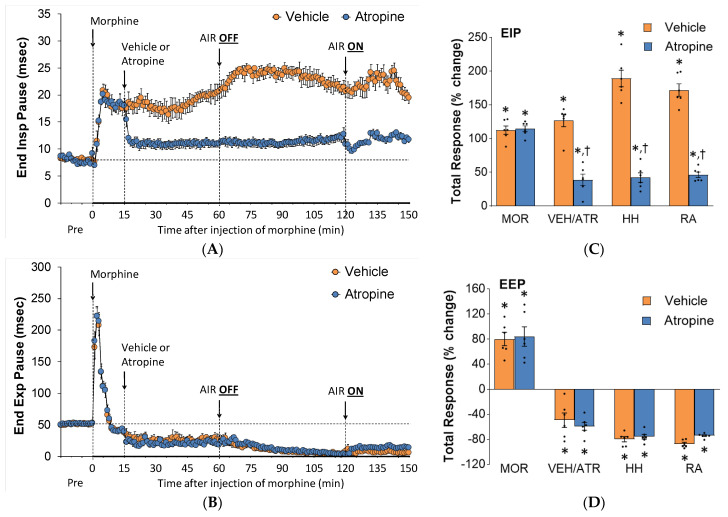
Values recorded for end inspiratory pause (End Insp Pause, EIP) (**A**) and end expiratory pause (End Exp Pause, EEP) (**B**) before (Pre), after injection of morphine (10 mg/kg, IV), and then subsequent injection of vehicle (VEH) or atropine (1 mg/kg, IV) followed by a hypoxic-hypercapnic gas challenge (AIR OFF) and return to room air (AIR ON). A summary of the total changes in EIP and EEP during each phase of the study are presented in (**C**,**D**), respectively (ATR, atropine; HH, hypoxic-hypercapnic gas challenge; RA, return to room air). Data are presented as mean ± SEM. There were 6 rats in each group. * *p* < 0.05, significant change from Pre-values. ^†^ *p* < 0.05, atropine versus vehicle.

As seen in Figure 3, morphine produced a substantial increase in EIP that was sustained throughout the protocol (panel A). Subsequent injection of atropine produced a prompt and sustained reduction in the morphine-induced increase in EIP (see panel C). In contrast, morphine elicited a substantial initial increase in EEP followed by a sustained fall to below baseline values of similar magnitude in both groups of rats (Panels B and D).

#### 2.2.4. Peak Inspiratory Flow (PIF), Peak Expiratory Flow (PEF) and PIF/PEF

**Figure 4:**
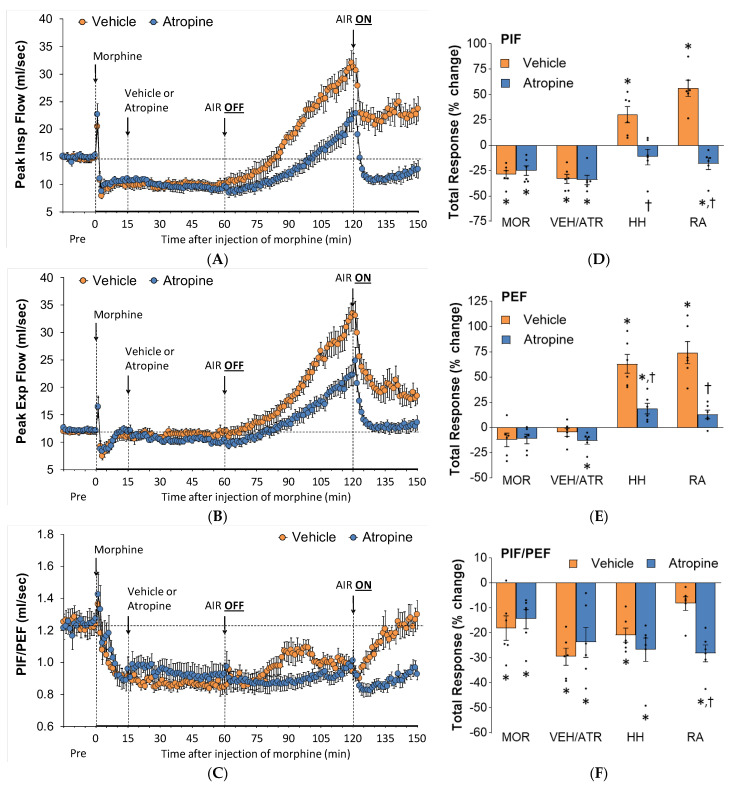
Values recorded for peak inspiratory flow (Peak Insp Flow, PIF) (**A**), peak expiratory flow (Peak Exp Flow, PEF) (**B**) and peak inspiratory flow/peak expiratory flow (PIF/PEF) (**C**) before (Pre), after injection of morphine (10 mg/kg, IV), and then subsequent injection of vehicle (VEH) or atropine (1 mg/kg, IV) followed by a hypoxic-hypercapnic gas challenge (AIR OFF) and return to room air (AIR ON). A summary of the total changes in PIF, PEF and PIF/PEF during each phase of the study are presented in (**D**–**F**), respectively (ATR, atropine; HH, hypoxic-hypercapnic gas challenge; RA, return to room air). Data are presented as mean ± SEM. There were 6 rats in each group. * *p* < 0.05, significant change from Pre-values. ^†^ *p* < 0.05, atropine versus vehicle.

As seen in Figure 4, morphine produced a sustained decrease in PIF that was not affected by the subsequent injection of atropine (panel A). The increases in PIF that occurred during the subsequent HH-challenge and upon return to room air were markedly diminished in the atropine-treated rats compared to the vehicle-treated rats. In contrast, neither morphine nor atropine exerted discernible effects on PEF (panel B). However, the increases in PEF during subsequent HH-challenge and upon return to room air were markedly diminished in atropine-treated rats compared to vehicle-treated rats. These changes in PIF and PEF resulted in substantial reductions in PIF/PEF throughout the protocol with the marked difference in PIF/PEF between the groups occurring upon return to room air. Summaries of the total changes in PIF, PEF and PIF/PEF (Panels B, D, F) highlight the atropine-mediated effects during the HH-challenge and upon return to room air.

#### 2.2.5. Rate of Achieving PEF (Rpef) and Expiratory Flow at 50% Expired TV (EF_50_)

**Figure 5:**
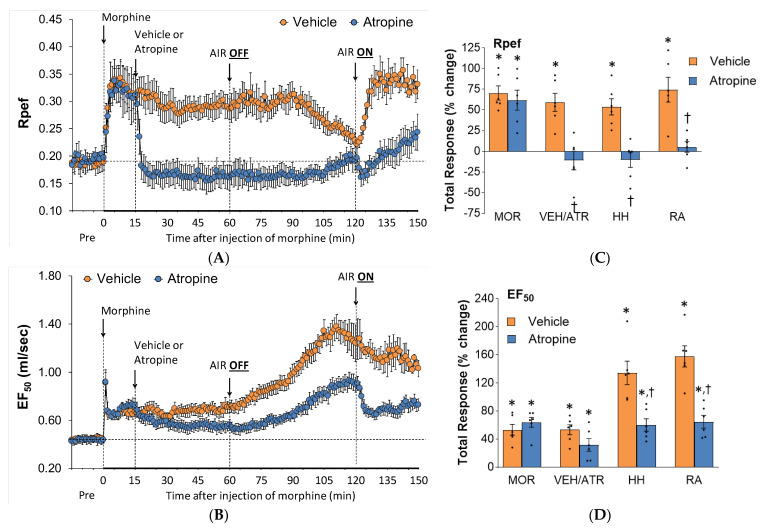
Values recorded for Rpef (**A**) and EF_50_ (**B**) before (Pre), after injection of morphine (10 mg/kg, IV), and then subsequent injection of vehicle (VEH) or atropine (1 mg/kg, IV) followed by a hypoxic-hypercapnic gas challenge (AIR OFF) and return to room air (AIR ON). A summary of the total changes in Rpef and EF_50_ during each phase of the study are presented in (**C**,**D**), respectively (ATR, atropine; HH, hypoxic-hypercapnic gas challenge; RA, return to room air). The data are presented as mean ± SEM. There were 6 rats in each group. * *p* < 0.05, significant change from Pre-values. ^†^ *p* < 0.05, atropine versus vehicle.

As seen in Figure 5, morphine produced a substantial increase in EIP that was sustained throughout the protocol (panel A). Subsequent injection of atropine produced a prompt and sustained reduction in this effect of morphine (see panel C). Morphine elicited a substantial increase in EF_50_ that was further increased during the HH-challenge and maintained upon return to room air (Panels B and D). Atropine elicited a sustained reduction in this effect of morphine and EF50 values remained lower in atropine-treated compared to vehicle-treated rats throughout the protocol (Panels B and D).

#### 2.2.6. Relaxation Time (RT), Expiratory Delay (Te-RT) and Apneic Pause [(Te/RT)-1]

**Figure 6:**
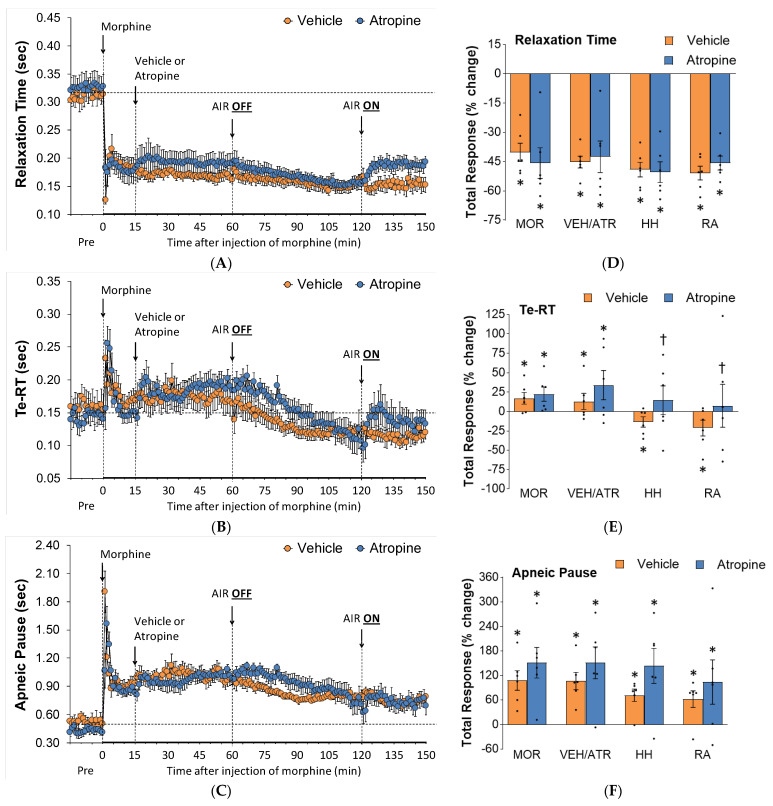
Values recorded for relaxation time (RT) (**A**), expiratory time—relaxation time (Te-RT) (**B**) and apneic pause (**C**) before (Pre), after injection of morphine (10 mg/kg, IV), and then subsequent injection of vehicle (VEH) or atropine (1 mg/kg, IV) followed by a hypoxic-hypercapnic gas challenge (AIR OFF) and return to room air (AIR ON). A summary of the total changes in RT, Te-RT and apneic pause during each phase of the study are presented in (**D**–**F**), respectively (ATR, atropine; HH, hypoxic-hypercapnic gas challenge; RA, return to room air). Data are presented as mean ± SEM. There were 6 rats in each group. * *p* < 0.05, significant change from Pre-values. ^†^ *p* < 0.05, atropine versus vehicle.

As seen in Figure 6, morphine produced a substantial decrease in RT that was sustained throughout the protocol with similar changes seen in the vehicle-treated and atropine-treated rats (panels A and D). Morphine produced a transient initial increase in Te-RT and these expiratory delay values remained slightly higher following the injection of vehicle and fell slightly below baseline values during HH-challenge and upon return to room air (panels B and D). Atropine blunted these falls in Te-RT during the HH-challenge and return to room air. Morphine produced sustained increases in apneic pause that were maintained during the HH-challenge and return to room air (panels C and F). Atropine had negligible effects on the morphine-induced increase in apneic pause and the apneic pause values recorded during the HH-challenge and return to room air (panels C and F).

#### 2.2.7. Inspiratory Drive (TV/Ti) and Expiratory Drive (TV/Te)

**Figure 7:**
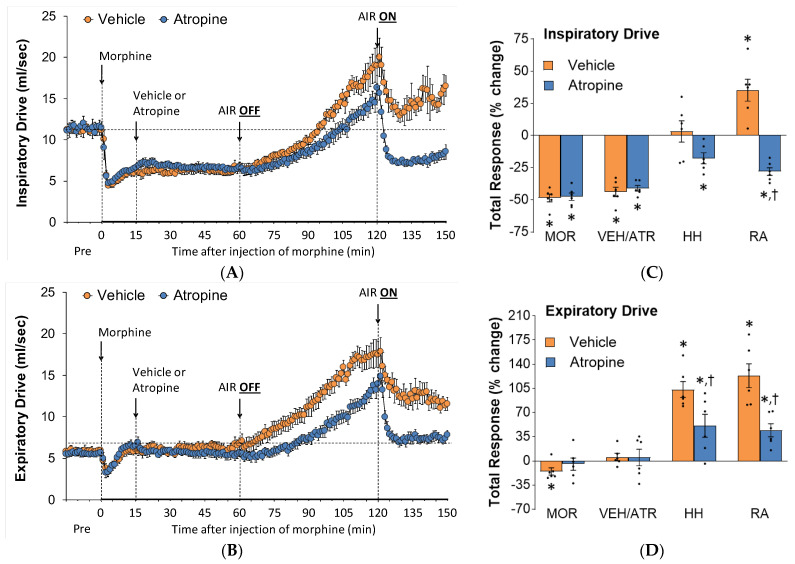
Values recorded for inspiratory drive (**A**) and expiratory drive (**B**) before (Pre), injection of morphine (10 mg/kg, IV), and then subsequent injection of vehicle (VEH) or atropine (1 mg/kg, IV) followed by a hypoxic-hypercapnic gas challenge (AIR OFF) and return to room air (AIR ON). A summary of the total changes in inspiratory drive and expiratory drive during each phase of the study are presented in (**C**,**D**), respectively (ATR, atropine; HH, hypoxic-hypercapnic gas challenge; RA, return to room air). Data are presented as mean ± SEM. There were 6 rats in each group. * *p* < 0.05, significant change from Pre-values. ^†^ *p* < 0.05, atropine versus vehicle.

As seen in Figure 7, morphine produced a sustained decrease in inspiratory drive that was not affected by the injection of atropine (panel A). The subsequent increases in inspiratory drive that occurred during HH-challenge and upon return to room air were diminished in atropine-treated rats compared to vehicle-treated rats. Morphine produced a relatively transient fall in expiratory drive and atropine produced minimal effects on this parameter (panel B). The increases in expiratory drive during subsequent HH-challenge and upon return to room air were markedly diminished in atropine-treated rats. Summaries of the total changes in inspiratory drive (Panel C) and expiratory drive (Panel D) highlight that atropine-markedly diminished the increases in these parameters during exposure to the HH-challenge and upon return to room air.

#### 2.2.8. Non-Eupneic Breathing Index (NEBI) and NEBI Corrected for Freq (NEBI/Freq)

**Figure 8:**
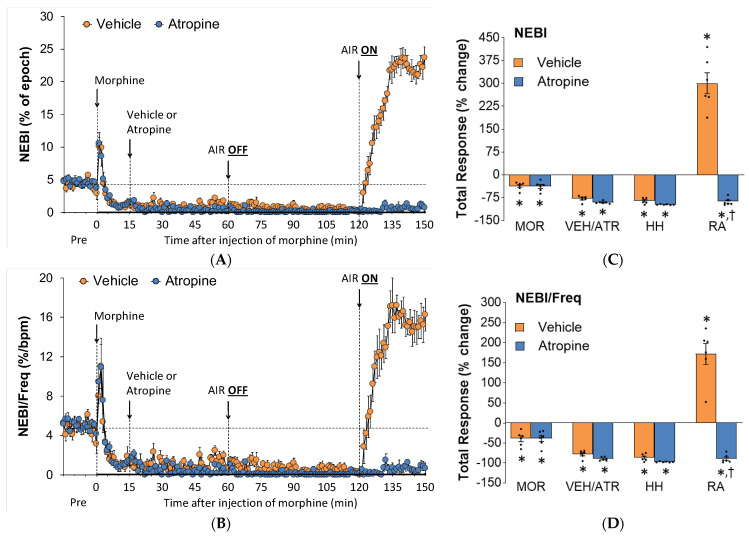
Values recorded for non-eupneic breathing index (NEBI) (**A**) and NEBI/Frequency of breathing (NEBI/Freq) (**B**) before (Pre), after injection of morphine (10 mg/kg, IV), and then subsequent injection of vehicle (VEH) or atropine (1 mg/kg, IV) followed by a hypoxic-hypercapnic gas challenge (AIR OFF) and return to room air (AIR ON). A summary of the total changes in NEBI and NEBI/Freq during each phase of the study are presented in (**C**,**D**), respectively (ATR, atropine; HH, hypoxic-hypercapnic gas challenge; RA, return to room air). Data are presented as mean ± SEM. There were 6 rats in each group. * *p* < 0.05, significant change from Pre-values. ^†^ *p* < 0.05, atropine versus vehicle.

As seen in Figure 8, morphine produced a transient increase followed by substantial and sustained decrease in NEBI and NEBI/Freq that was not affected by the injection of atropine or the HH-challenge (panels A and B). The marked and sustained increases in NEBI and NEBI/Freq upon return to room air were absent in the atropine-treated rats. Summaries of the total changes in NEBI (Panel C) and NEBI/Freq (Panel D) highlight that atropine markedly eliminated the increases in these parameters upon return to room air (i.e., these values remained suppressed).

### 2.3. Righting Reflex

The loss of righting reflex took 73.1 ± 6.2 min to recover following the injection of morphine (10 mg/kg, IV) in vehicle-treated rats. The righting reflex in morphine-treated rats that received an injection of atropine (1 mg/kg, IV) recovered more quickly (45.6 ± 4.4 min) than in vehicle-treated rats (*p* < 0.05). All rats returned to normal grooming activities and no other (unusual) behavioral differences were observed between the groups.

## 3. Discussion

### 3.1. Ventilatory Responses Elicited by Morphine

The present study demonstrates that the intravenous injection of morphine (10 mg/kg, IV) produced an array of ventilatory responses in adult male Sprague Dawley rats that were consistent with those we have reported previously [49,50,51,52,53,54,55,56]. In brief, this dose of morphine had minimal effects of Freq but pronounced and sustained effects on tidal volume (and therefore minute ventilation). However, the lack of effect of morphine on frequency of breathing belies the fact that the opioid markedly elevated inspiratory time and EIP while decreasing expiratory time and EEP. As such, despite minimal effects of morphine on frequency of breathing per se, it is evident that the opioid has pronounced and opposing effects on processes controlling inspiratory and expiratory timing. The inhibitory effect of morphine on PIF was also in stark contrast to the observed increases in PEF, Rpef and EF_50_ elicited by the opioid. This clearly shows that morphine is also able to differentially affect the processes involved in the neural/musculoskeletal control of inspiration and expiration [28]. The morphine-induced enhancement of expiratory delay and apneic pause highlights that this opioid is similar to fentanyl in its ability to adversely affect upper airway dynamics [25,26,27,28]. Finally, the finding that morphine elicited a substantial initial increase in NEBI and NEBI/Freq is consistent with opioids destabilizing eupneic breathing [56] whereas the dramatic subsequent fall in these indices would unexpectedly suggest that morphine actually eliminates non-eupneic events, which in these rats, are most commonly apneas and disordered breaths [25,26,27,28].

### 3.2. Ventilatory Responses Elicited by Atropine in Morphine-Treated Rats

Subsequent injection of atropine (1 mg/kg, IV) in the morphine-treated rats dramatically reduced the morphine-induced increases in EIP, Rpef and EF_50_, whereas atropine did not affect frequency of breathing, tidal volume, minute ventilation, inspiratory and expiratory times, end expiratory pause, peak inspiratory or expiratory flows, relaxation time, expiratory delay, apneic pause, inspiratory and expiratory drives and non-eupneic breathing index. It appears that muscarinic receptor mechanisms are vital in the ability of morphine to lengthen the pause before expiration (i.e., the increase in EIP), noting that (a) cholinergic muscarinic transmission contributes to excitation of parafacial respiratory group (pFRG) neurons and promotes both active recruitment of abdominal muscles and active expiratory flow [36], (b) pontine cholinergic mechanisms play vital roles in respiratory regulation [58], (c) the transition from inspiration to expiration is a phenomenon depending on active interplay between intrinsic neural networks in the pons and input from slowly adapting pulmonary stretch receptors [59], and (d) these systems are influenced by the intermediate reticular nucleus that helps to co-ordinate post-inspiratory activity, swallowing, and respiratory-sympathetic coupling [60]. It also appears that cholinergic mechanisms are vital to the yet-to-be-defined processes by which morphine causes a more rapid attaining of PEF (i.e., Rpef) while not enhancing PEF per se [61]. Our companion manuscript [43] reports that atropine (1 mg/kg, IV) elicits profound effects on ventilatory parameters in naïve adult male Sprague Dawley rats. Supplemental Table S4 provides a qualitative comparison of the effects of atropine in these naïve rats and those of the present study. It is evident that morphine dramatically reduces the impact of atropine on ventilatory parameters by mechanisms in ways not necessarily due to complications related to changes in baseline values. For example, atropine causes a pronounced increase in the frequency of breathing in naïve rats, whereas it has no effect on morphine-treated rats, noting that morphine did not change baseline frequency values. It would therefore seem that morphine must curtail cholinergic signaling in neural circuits that control the frequency of breathing. The general lack of effect of atropine on the morphine-induced changes in ventilatory parameters in freely moving rats are not consistent with evidence of the activation of muscarinic receptor-dependent mechanisms in the medulla oblongata reverse fentanyl-induced respiratory depression in isoflurane-anesthetized rats [62]. From these latter findings, it would follow that blockade of muscarinic receptors would augment the ventilatory depressant effects of opioids including morphine.

### 3.3. Ventilatory Responses During Exposure to a HH-Challenge

The mechanisms responsible for the ventilatory responses to HH-challenges [63] involve carotid body and brainstem signal transduction processes [64,65] and the interplay between these two stimuli has received attention [66,67,68]. There is no information at present regarding potential roles of cholinergic neuronal-muscarinic receptor systems in expression of ventilatory responses to HH-challenge. However, Lydic et al. [69] reported that cholinergic mechanisms in the medial prefrontal cortex influence state-dependent ventilatory response to hypercapnia in cats via muscarinic receptor activation. Based on elegant electrophysiological recordings of neurons in the brainstem respiratory chemosensitive area of chloralose-urethane anesthetized cats, Trouth et al. [70] suggested that the endogenous opiates are involved in the central regulation of respiration by interactions with CO_2_-sensitive cholinergic structures located at the caudal ventral medullary surface. As expected, HH-challenge elicited robust changes in ventilatory parameters [63]. A key finding was that many of these responses were substantially affected by atropine whereas others were not, whereas HH-challenge-induced increase in TV was markedly reduced in atropine-treated rats the corresponding increases in Freq (and associated changes in Ti and Te) were not affected by the muscarinic receptor antagonist. In addition, the increases in PIF, PEF, EF_50_, inspiratory drive and expiratory drive were markedly reduced by atropine whereas changes in relaxation time, expiratory delay and NEBI were not affected with pronounced effects of morphine and atropine on Rpef, making the interpretation of the lack of effects of the HH-challenge difficult to interpret. To our knowledge this is the first clear example of a study in which a differing role for muscarinic receptor signaling processes in ventilatory signaling responses to HH-challenge has been suggested. The involvement of brain and carotid body cholinergic mechanisms in these HH-challenge responses remain to be determined.

### 3.4. Ventilatory Responses Following Return to Room Air

The neurophysiological and neurochemical mechanisms responsible for the ventilatory responses that occur upon return to room air following hypoxic, hypercapnic and HH-challenges have received some attention, and it has been established that the carotid body-carotid sinus nerve complex is vital and that redox status and nitric oxide synthase and S-nitrosothiols also play important roles [71]. Atropine had dramatic effects on some of the ventilatory responses that occurred upon return to room air after the HH-challenge. In particular, the changes in Freq (and Ti), TV, MV, PIF, PEF, Rpef, inspiratory and expiratory drives observed in vehicle-treated rats were markedly reduced in the atropine-treated rats. Notably, the substantial increase in non-eupneic breathing index (NEBI) was absent in atropine-treated rats. The increase in NEBI was likely due to increases in apneic episodes and frequency of disordered breaths, in particular [71], and as such it is evident that cholinergic signaling via muscarinic receptors plays a major role in the destabilization of breathing during the return to room air phase following HH-challenge. Whether this increase in NEBI involves the destabilization of neurotransmission within the carotid bodies and or respiratory control centers in the brain remains to be established.

### 3.5. Role of Muscarinic Receptors in Morphine-Induced Effects on Righting Reflex

Our studies show that atropine markedly reduced the time for the morphine-treated rats to regain their righting reflex. These findings are not necessarily easy to interpret since it would be expected that if the sedative effects of morphine were simply due to the neural release of acetylcholine and subsequent activation of muscarinic receptors, then the injection of atropine would be expected to arouse the rats more or less immediately rather than shortening the duration of sedation from 73.1 ± 6.2 min to 45.6 ± 4.4 min. It appears that the activation of muscarinic receptors becomes increasingly important to the maintenance rather than the initiation of morphine-induced sedation. The ability of atropine to ameliorate the sedative actions of morphine in rats is consistent with evidence that (a) atropine ameliorates morphine plus nicotine-induced catalepsy in mice [72], (b) muscarinic receptor mechanisms within the basolateral amygdala are involved in the acquisition of morphine-induced place preference in rats [73], (c) muscarinic receptors within the hippocampus [74] and the ventral tegmentum [75] are involved in mediating the rewarding effects of morphine in rats, (d) codeine produces muscarinic receptor-mediated analeptic effect in rats and rabbits [76], and (e) fentanyl produces muscarinic receptor-mediated analeptic and EEG arousal effects in rats [77].

### 3.6. Conclusions

The primary conclusions of this study are that (a) atropine affects specific aspects of the effects of morphine on ventilatory parameters (EIP, Rpef, EF_50_) while not affecting core ventilatory parameters such as Freq, TV or MV, (b) atropine has substantial effects on the ventilatory responses, including TV, that occur during and following a HH-challenge, and (c) atropine reduces the sedative actions of morphine. Regarding the recovery of the righting reflex following morphine administration, it is important to note the vehicle group required 73.1 ± 6.2 min to recover their righting reflex, whereas this recovery time was shortened to 45.6 ± 4.4 min in the atropine group. Since the HH-challenge commenced 60 min after morphine administration, it appears that the rats in the vehicle group were in a deeper state of sedation at the onset of the HH-challenge than those in the atropine group, which were likely closer to a full recovery of alertness. The mitigation of morphine sedation by atropine would, in theory, enhance the ventilatory responses to HH-challenges. However, our findings that the increases in TV, MV, PIF, PEF, and inspiratory and expiratory drives during the HH-challenges and subsequent return to room air phase were diminished in atropine-treated rats suggests that the activation of muscarinic receptors play a direct role in the expression of the ventilatory effects of HH-challenge and that these effects countermand any influence of the lower degree of sedation. Major limitations of this study pertain to (a) the need to perform studies to determine to which of the five muscarinic receptors (M1-M5) blocked by atropine are involved in the responses described above, (b) the necessity to study female rats because of sex differences in neurobiological/neurochemical processes controlling breathing, and (c) the need to study a peripherally restricted muscarinic receptor antagonist, such as methyl-atropine [78] to determine to what extent peripheral (e.g., carotid bodies) and central muscarinic receptor systems are involved in the effects described above. The findings of the present study help to clarify the role of muscarinic receptors in the ability of L-CYSee to overcome the ventilatory depressant effects of morphine in freely moving rats [43]. Future studies will focus on understanding the role of peripheral and central M1-M5 muscarinic receptors in the respiratory depressant effects of morphine in male and in female rats. Our studies demonstrating the focused role of muscarinic receptors in aspects morphine-induced OIRD will lead to more expansive studies to understand their involvement in the roles of endoplasmic reticulum stress [79] and glutamate receptors [80] in the development of morphine tolerance and in neural [81] and hepatic injuries [82]. A limitation of our present study and companion manuscript [43] is that atropine was administered after morphine, and follow-up studies will determine the effects of pretreatment with atropine on the ventilatory responses to morphine to help further address the role of muscarinic receptors in the actions of morphine and influences such as behavioral state or depth of sedation. While the HH-challenge allows the observation of ventilatory phenotypes under physiological stress (increasingly hypoxic and hypercapnic environment), time-series recordings of ventilatory parameters alone cannot directly substantiate alterations in central or peripheral chemoreflex sensitivity. A more rigorous analysis of respiratory physiology would correlate the ventilatory response with the intensity of the CO_2_/O_2_ stimulus, for instance, by calculating the slope of the hypercapnic or hypoxic ventilatory response rather than just comparing mean values or percentage changes between the “AIR OFF” and “AIR ON” phases. As such, our data only support the conclusion that systemic atropine altered plethysmographic ventilatory responses during and after the HH rebreathing protocol. To address these issues, we are currently performing studies in which we are concomitantly recording plethysmography chamber concentrations of O_2_ and CO_2_ and ventilatory parameters to establish the direct relationships between the changes chamber O_2_/CO_2_ and each ventilatory parameter during the HH-challenge with and without morphine or atropine. Finally, the strengths and limitations of the whole body plethysmography techniques used in the present study should be recognized and have been reviewed in great detail by others such as Stephenson and Gucciardi [83] and Zysman-Colman and Lands [84].

## 4. Materials and Methods

### 4.1. Permissions, Rats, Surgical Procedures

All studies were carried out in strict accordance with the NIH Guide for Care and Use of Laboratory Animals (NIH Publication No. 80–23) revised in 1996, and in strict compliance with the ARRIVE (Animal Research: Reporting of In Vivo Experiments) guidelines (https://www.inotiv.com/, accessed on 10 December 1997). All experimental protocols were approved by the Animal Care and Use Committees of the University of Virginia, Galleon Pharmaceuticals, Inc., and Case Western Reserve University. Adult male Sprague Dawley rats from Harlan Industries (Madison, WI, USA) were given 6 days of recovery from transportation and then implanted with jugular vein catheters under 2–3% isoflurane anesthesia [44,45,46,47,48]. The catheters were flushed with 0.3 mL of phosphate-buffered saline (0.1 M, pH 7.4) 3–4 h before starting the protocol. Morphine sulfate was from Baxter Healthcare Corporation (Deerfield, IL, USA) and provided to us by our Animal Resources Centers. Atropine sulfate hydrate (A0257-5G) was from Sigma-Aldrich (St. Louis, MO, USA). The doses of morphine sulfate (10 mg/kg, IV) and atropine sulfate hydrate (1 mg/kg, IV) are expressed in terms of base compound (morphine and atropine, respectively). The experiments were performed in a quiet room with relative humidity of 49 ± 2% and temperature of 21.3 ± 0.2 °C. Plethysmography sessions were done by an investigator who administered the vehicle and test drugs. The syringes with vehicle or test drug were made up by another investigator so that the person doing the study was blinded to the protocol. Data files from each experiment were collated and analyzed by another investigator. These studies were performed in 2 separate cohorts of vehicle-treated (3 and 3 rats) or atropine-treated rats (3 and 3 rats) done 4 months apart.

### 4.2. Protocols for Measurement of Ventilatory Parameters

Ventilatory parameters were recorded continuously in unrestrained freely moving rats via whole body plethysmography system (PLY3223; Data Sciences International, St. Paul, MN, USA) as detailed previously [25,26,27,28]. Directly recorded and derived parameters are defined in Supplemental Table S2. These parameters are: Freq, frequency of breathing; TV, tidal volume; MV, minute ventilation; Ti, inspiratory time; Te, expiratory time; Te/Ti (respiratory quotient); EIP, end inspiratory pause; EEP, end expiratory pause; PIF, peak inspiratory flow; PEF, peak expiratory flow; PEF/PIF (flow balance); Rpef, rate of achieving PEF; EF_50_, expiratory flow at 50% expired TV; RT, relaxation time; expiratory delay, Te-RT; AP, apneic pause, (Te/RT)-1; TV/TI, inspiratory drive; TV/Te, expiratory drive; NEBI, non-eupneic breathing index; and NEBI/Freq, NEBI corrected for the frequency of breathing. A diagram of the relationships between directly recorded parameters and, especially, the relationship between Te and RT are provided in Supplemental Figure S1 [25,26,27,28]. On the day of the study, each rat was placed in an individual plethysmography chamber and allowed at least 60 min to acclimatize so that resting (baseline, pre) ventilatory parameters were accurately defined. Protocol: Two groups of rats received an injection of morphine (10 mg/kg, IV) and after 15 min, one group received an injection of vehicle (saline) and the other received an injection of atropine (1.0 mg/kg, IV). Forty-five min later, all rats were exposed to a hypoxic-hypercapnic (HH) challenge (rebreathing method) as described previously [54] and then re-exposed to room air for 30 min. The body weights of the two groups of rats were similar to one another (Supplemental Table S3) and so volume parameters (e.g., TV, PIF, PEF, EF_50_) were not corrected for body weight. FinePointe (DSI) software constantly corrected digitized ventilatory values originating from raw waveforms for alterations in chamber humidity and temperature. Pressure changes associated with respiratory waveforms were converted to volumes (e.g., TV, PIF, PEF, EF_50_) using proprietary software algorithms. Factoring in chamber humidity and temperature, cycle analyzers filtered the acquired signals, and FinePointe algorithms generated box flow data that identified a waveform segment as an acceptable breath. From that data, maximum and minimum values were then determined. Flows at this time were box flow signals and from these, maximum and minimum and box flow values were determined and multiplied by a compensation factor provided by the selected algorithm to produce TV, PIF, PEF and EF_50_ values used to determine non-eupneic breathing events expressed as non-eupneic breathing index (NEBI) and expressed as percentage of non-eupneic breathing events per recording epoch [25,26,27,28].

### 4.3. Righting Reflex as an Index of Morphine-Induced Sedation

Adult male Sprague Dawley rats were used to evaluate the effects of atropine on the duration of the loss of the righting reflex (i.e., inability to stand on all 4 legs) elicited by morphine [25,26,27,28]. The rats were put into individual open plastic chambers to determine the length of time they took to recover from the morphine-induced loss of the righting reflex. The jugular vein catheter was connected to tubing connected to a syringe to inject drugs. The rats were given 90 min to acclimatize. The time when the rat stood on all four paws for at least 12 s after injection of morphine was taken as the time of recovery of the righting reflex [25,26,27,28]. One group of rats (n = 9, 337 ± 2 g) received morphine (10 mg/kg, IV) and after 15 min they received an injection of vehicle (saline). A second group (n = 9, 336 ± 3 g) received morphine (10 mg/kg, IV), and an injection of atropine (1.0 mg/kg, IV) 15 min later. Recovery of the righting reflex was determined by two observers blind to the protocols.

### 4.4. Data Analyses

All data are presented as mean ± SEM and were analyzed by one-way and two-way ANOVA and Bonferroni corrections for multiple comparisons between means using the error mean square terms generated by ANOVA analyses [25,26,27,28]. A *p* < 0.05 value was the initial level of significance that was modified according to the number of between-mean comparisons [25,26,27,28]. The modified *t-*statistic for 2 groups for instance is t = (mean group 1 − mean group 2)/[s × (1/n_1_ + 1/n_2_)^1/2^] where s^2^ = mean square within groups term from the ANOVA and n_1_ and n_2_ are the number of rats in each group being compared. Statistics were done with GraphPad Prism (version 9.5.1) software (GraphPad Software, Inc., La Jolla, CA, USA).

## Data Availability

The original contributions presented in this study are included in the article and Supplementary Materials. Further inquiries can be directed to the corresponding author.

## Supplementary Materials

The following supporting information can be downloaded at: https://www.mdpi.com/article/10.3390/ph19071101/s1 Table S1: Updated list of the different classes of drugs that provide potential mechanisms of action in reversing opioid-induced respiratory depression (OIRD) and show promise in providing effective therapeutics for OIRD, Table S2: Definition of ventilatory parameters. Table S3: Baseline ventilatory parameters. Table S4: Comparison of the effects of atropine in naïve and morphine-treated rats. Figure S1: Relationships between peak inspiratory flow (PIF), peak expiratory flow (PEF), relaxation time (RT) and expiratory time (Te).

## Abbreviations

OIRD, opioid-induced respiratory depression; NMDA, N-methyl-D-aspartate; AMPA α-amino-3-hydroxy-5-methyl-4-isoxazolepropionic acid receptor; HH, hypoxic-hypercapnic; Freq, frequency of breathing; TV, tidal volume; MV, minute ventilation; Ti, inspiratory time; Te, expiratory time; Te/Ti (respiratory quotient); EIP, end inspiratory pause; EEP, end expiratory pause; PIF, peak inspiratory flow; PEF, peak expiratory flow; PEF/PIF (flow balance); Rpef, rate of achieving PEF; EF_50_, expiratory flow at 50% expired TV; RT, relaxation time; expiratory delay, Te-RT; AP, apneic pause, (Te/RT)-1; TV/TI, inspiratory drive; TV/Te, expiratory drive; NEBI, non-eupneic breathing index; and NEBI/Freq, NEBI corrected for the frequency of breathing. L-CYSee, L-cysteine ethyl ester.

## References

[1] Dahan A., Aarts L., Smith T.W. (2010). Incidence, Reversal, and Prevention of Opioid-induced Respiratory Depression. Anesthesiology.

[2] Boom M., Niesters M., Sarton E., Aarts L., Smith T.W., Dahan A. (2012). Non-analgesic effects of opioids: Opioid-induced respiratory depression. Curr. Pharm. Des..

[3] van der Schier R., Roozekrans M., van Velzen M., Dahan A., Niesters M. (2014). Opioid-induced respiratory depression: Reversal by non-opioid drugs. F1000Prime Rep..

[4] Algera M.H., Kamp J., van der Schrier R., van Velzen M., Niesters M., Aarts L., Dahan A., Olofsen E. (2019). Opioid-induced respiratory depression in humans: A review of pharmacokinetic-pharmacodynamic modelling of reversal. Br. J. Anaesth..

[5] Jonkman K., van Rijnsoever E., Olofsen E., Aarts L., Sarton E., van Velzen M., Niesters M., Dahan A. (2018). Esketamine counters opioid-induced respiratory depression. Br. J. Anaesth..

[6] Dai W., Xiao D., Gao X., Zhou X.B., Fang T.Y., Yong Z., Su R.B. (2017). A brain-targeted ampakine compound protects against opioid-induced respiratory depression. Eur. J. Pharmacol..

[7] Dai W., Gao X., Xiao D., Li Y.L., Zhou X.B., Yong Z., Su R.B. (2019). The Impact and Mechanism of a Novel Allosteric AMPA Receptor Modulator LCX001 on Protection Against Respiratory Depression in Rodents. Front. Pharmacol..

[8] Sahibzada N., Ferreira M., Wasserman A.M., Taveira-DaSilva A.M., Gillis R.A. (2000). Reversal of morphine-induced apnea in the anesthetized rat by drugs that activate 5-hydroxytryptamine_1A_ receptors. J. Pharmacol. Exp. Ther..

[9] Manzke T., Guenther U., Ponimaskin E.G., Haller M., Dutschmann M., Schwarzacher S., Richter D.W. (2003). 5-HT4a receptors avert opioid-induced breathing depression without loss of analgesia. Science.

[10] Vonhof S., Sirén A.L. (1991). Reversal of μ-opioid-mediated respiratory depression by α2-adrenoceptor antagonism. Life Sci..

[11] Ballanyi K., Lalley P.M., Hoch B., Richter D.W. (1997). cAMP-dependent reversal of opioid- and prostaglandin-mediated depression of the isolated respiratory network in newborn rats. J. Physiol..

[12] Lalley P.M. (2004). Dopamine1 receptor agonists reverse opioid respiratory network depression, increase CO_2_ reactivity. Respir. Physiol. Neurobiol..

[13] Lalley P.M. (2005). D1-dopamine receptor agonists prevent and reverse opiate depression of breathing but not antinociception in the cat. Am. J. Physiol. Regul. Integr. Comp. Physiol..

[14] Sia R.L., Zandstra D.F. (1981). 4-Aminopyridine reversal of fentanyl-induced respiratory depression in normocapnic and hypercapnic patients. Br. J. Anaesth..

[15] Roozekrans M., van der Schrier R., Okkerse P., Hay J., McLeod J.F., Dahan A. (2014). Two studies on reversal of opioid-induced respiratory depression by BK-channel blocker GAL021 in human volunteers. Anesthesiology.

[16] Roozekrans M., Olofsen E., van der Schrier R., van Gerven J., Peng S., McLeod J., Dahan A. (2015). Reversal of opioid-induced respiratory depression by BK-channel blocker GAL021: A pharmacokinetic-pharmacodynamic modeling study in healthy volunteers. Clin. Pharmacol. Ther..

[17] Golder F.J., Dax S., Baby S.M., Gruber R., Hoshi T., Ideo C., Kennedy A., Peng S., Puskovic V., Ritchie D. (2015). Identification and Characterization of GAL-021 as a Novel Breathing Control Modulator. Anesthesiology.

[18] Wei A.D., Ramirez J.M. (2019). Presynaptic Mechanisms and KCNQ Potassium Channels Modulate Opioid Depression of Respiratory Drive. Front. Physiol..

[19] Liang X., Yong Z., Su R. (2018). Inhibition of protein kinase A and GIRK channel reverses fentanyl-induced respiratory depression. Neurosci. Lett..

[20] Kasaba T., Takeshita M., Takasaki M. (1997). The effects of caffeine on the respiratory depression by morphine. Masui.

[21] Kimura S., Ohi Y., Haji A. (2015). Blockade of phosphodiesterase 4 reverses morphine-induced ventilatory disturbance without loss of analgesia. Life Sci..

[22] Hutchinson M.R., Northcutt A.L., Chao L.W., Kearney J.J., Zhang Y., Berkelhammer D.L., Loram L.C., Rozeske R.R., Bland S.T., Maier S.F. (2008). Minocycline suppresses morphine-induced respiratory depression, suppresses morphine-induced reward, and enhances systemic morphine-induced analgesia. Brain Behav. Immun..

[23] Owen M.D., Unal C.B., Callahan M.F., Trivedi K., York C., Millington W.R. (2000). Glycyl-glutamine inhibits the respiratory depression, but not the antinociception, produced by morphine. Am. J. Physiol..

[24] Boghosian J.D., Luethy A., Cotton J.F. (2018). Intravenous and Intratracheal Thyrotropin Releasing Hormone and Its Analog Taltirelin Reverse Opioid-Induced Respiratory Depression in Isoflurane Anesthetized Rats. J. Pharmacol. Exp. Ther..

[25] Seckler J.M., Grossfield A., May W.J., Getsy P.M., Lewis S.J. (2022). Nitrosyl factors play a vital role in the ventilatory depressant effects of fentanyl in unanesthetized rats. Biomed. Pharmacother..

[26] Getsy P.M., Young A.P., Bates J.N., Baby S.M., Seckler J.M., Grossfield A., Hsieh Y.H., Lewis T.H.J., Jenkins M.W., Gaston B. (2022). S-nitroso-L-cysteine stereoselectively blunts the adverse effects of morphine on breathing and arterial blood gas chemistry while promoting analgesia. Biomed. Pharmacother..

[27] Getsy P.M., Baby S.M., May W.J., Young A.P., Gaston B., Hodges M.R., Forster H.V., Bates J.N., Wilson C.G., Lewis T.H.J. (2022). D-Cysteine Ethyl Ester Reverses the Deleterious Effects of Morphine on Breathing and Arterial Blood-Gas Chemistry in Freely-Moving Rats. Front. Pharmacol..

[28] Baby S.M., May W.J., Young A.P., Wilson C.G., Getsy P.M., Coffee G.A., Lewis T.H.J., Hsieh Y.H., Bates J.N., Lewis S.J. (2024). L-cysteine ethylester reverses the adverse effects of morphine on breathing and arterial blood-gas chemistry while minimally affecting antinociception in unanesthetized rats. Biomed. Pharmacother..

[29] Elmalem E., Chorev M., Weinstock M. (1991). Antagonism of morphine-induced respiratory depression by novel anticholinesterase agents. Neuropharmacology.

[30] Tsujita M., Sakuraba S., Kuribayashi J., Hosokawa Y., Hatori E., Okada Y., Kashiwagi M., Takeda J., Kuwana S. (2007). Antagonism of morphine-induced central respiratory depression by donepezil in the anesthetized rabbit. Biol. Res..

[31] Sakuraba S., Tsujita M., Arisaka H., Takeda J., Yoshida K., Kuwana S. (2009). Donepezil reverses buprenorphine-induced central respiratory depression in anesthetized rabbits. Biol. Res..

[32] Ren J., Ding X., Greer J.J. (2019). Activating α4β2 Nicotinic Acetylcholine Receptors Alleviates Fentanyl-induced Respiratory Depression in Rats. Anesthesiology.

[33] Ren J., Ding X., Greer J.J. (2020). Countering Opioid-induced Respiratory Depression in Male Rats with Nicotinic Acetylcholine Receptor Partial Agonists Varenicline and ABT 594. Anesthesiology.

[34] Kubin L., Fenik V. (2004). Pontine cholinergic mechanisms and their impact on respiratory regulation. Respir. Physiol. Neurobiol..

[35] Bonis J.M., Neumueller S.E., Krause K.L., Kiner T., Smith A., Marshall B.D., Qian B., Pan L.G., Forster H.V. (2010). A role for the Kolliker-Fuse nucleus in cholinergic modulation of breathing at night during wakefulness and NREM sleep. J. Appl. Physiol..

[36] Boutin R.C., Alsahafi Z., Pagliardini S. (2017). Cholinergic modulation of the parafacial respiratory group. J. Physiol..

[37] Moreira T.S., Sobrinho C.R., Falquetto B., Oliveira L.M., Lima J.D., Mulkey D.K., Takakura A.C. (2021). The retrotrapezoid nucleus and the neuromodulation of breathing. J. Neurophysiol..

[38] Biancardi V., Yang X., Ding X., Passi D., Funk G.D., Pagliardini S. (2023). Cholinergic projections to the preBötzinger complex. J. Comp. Neurol..

[39] Eyzaguirre C., Zapata P. (1968). The release of acetylcholine from carotid body tissues. Further study on the effects of acetylcholine and cholinergic blocking agents on the chemosensory discharge. J. Physiol..

[40] Fidone S., Sato A., Eyzaguirre C. (1968). Acetylcholine activation of carotid body chemoreceptor A fibers. Brain Res..

[41] Nurse C.A., Zhang M. (1999). Acetylcholine contributes to hypoxic chemotransmission in co-cultures of rat type 1 cells and petrosal neurons. Respir. Physiol..

[42] Kim D.K., Prabhakar N.R., Kumar G.K. (2004). Acetylcholine release from the carotid body by hypoxia: Evidence for the involvement of autoinhibitory receptors. J. Appl. Physiol..

[43] Getsy P.M., May W.J., Young A.P., Baby S.M., Coffee G.A., Forster H.V., Hodges M.R., Qiu Y., Cheng F., Bates J.N. (2026). L-cysteine ethyl ester may activate muscarinic receptor signaling processes to overcome morphine-induced respiratory depression in freely-moving rats. Pharmaceuticals.

[44] Svoboda J., Popelikova A., Stuchlik A. (2017). Drugs Interfering with Muscarinic Acetylcholine Receptors and Their Effects on Place Navigation. Front. Psychiatry.

[45] Zhang W., Basile A.S., Gomeza J., Volpicelli L.A., Levey A.I., Wess J. (2022). Characterization of central inhibitory muscarinic autoreceptors by the use of muscarinic acetylcholine receptor knock-out mice. J. Neurosci..

[46] Picciotto M.R., Higley M.J., Mineur Y.S. (2012). Acetylcholine as a neuromodulator: Cholinergic signaling shapes nervous system function and behavior. Neuron.

[47] Jiang S., Li Y., Zhang C., Zhao Y., Bu G., Xu H., Zhang Y.W. (2014). M1 muscarinic acetylcholine receptor in Alzheimer’s disease. Neurosci. Bull..

[48] Lakstygal A.M., Kolesnikova T.O., Khatsko S.L., Zabegalov K.N., Volgin A.D., Demin K.A., Shevyrin V.A., Wappler-Guzzetta E.A., Kalueff A.V. (2019). DARK Classics in Chemical Neuroscience: Atropine, Scopolamine, and Other Anticholinergic Deliriant Hallucinogens. ACS Chem. Neurosci..

[49] Dinger B.G., Hirano T., Fidone S.J. (1986). Autoradiographic localization of muscarinic receptors in rabbit carotid body. Brain Res..

[50] Dasso L.L., Buckler K.J., Vaughan-Jones R.D. (1997). Muscarinic and nicotinic receptors raise intracellular Ca^2+^ levels in rat carotid body type I cells. J. Physiol..

[51] Shirahata M., Hirasawa S., Okumura M., Mendoza J.A., Okumura A., Balbir A., Fitzgerald R.S. (2004). Identification of M1 and M2 muscarinic acetylcholine receptors in the cat carotid body chemosensory system. Neuroscience.

[52] Bairam A., Joseph V., Lajeunesse Y., Kinkead R. (2006). Developmental pattern of M1 and M2 muscarinic gene expression and receptor levels in cat carotid body, petrosal and superior cervical ganglion. Neuroscience.

[53] Ortiz F.C., Varas R. (2010). Muscarinic modulation of TASK-like background potassium channel in rat carotid body chemoreceptor cells. Brain Res..

[54] May W.J., Henderson F., Gruber R.B., Discala J.F., Young A.P., Bates J.N., Palmer L.A., Lewis S.J. (2013). Morphine has latent deleterious effects on the ventilatory responses to a hypoxic-hypercapnic challenge. Open J. Mol. Integr. Physiol..

[55] Matthew C.B., Thomas G.J., Hubbard R.W., Francesconi R.P. (1988). Intramuscular and intravenous atropine: Comparison of effects in the heat-stressed rat. Aviat. Space Environ. Med..

[56] Pagniez F., Valentin J.P., Vieu S., Colpaert F.C., John G.W. (1998). Pharmacological analysis of the haemodynamic effects of 5-HT1B/D receptor agonists in the normotensive rat. Br. J. Pharmacol..

[57] Nakajima Y., Tsujimura T., Tsutsui Y., Chotirungsan T., Kawada S., Dewa N., Magara J., Inoue M. (2023). Atropine facilitates water-evoked swallows via central muscarinic receptors in anesthetized rats. Am. J. Physiol. Gastrointest. Liver Physiol..

[58] Sergeeva L.I., Kuz’mina V.E. (1994). Participation of cholinergic systems in the bulbar mechanisms of the regulation of breathing. Neurosci. Behav. Physiol..

[59] Bonham A.C. (1995). Neurotransmitters in the CNS control of breathing. Respir. Physiol..

[60] Toor R.U.A.S., Sun Q.J., Kumar N.N., Le S., Hildreth C.M., Phillips J.K., McMullan S. (2019). Neurons in the Intermediate Reticular Nucleus Coordinate Postinspiratory Activity, Swallowing, and Respiratory-Sympathetic Coupling in the Rat. J. Neurosci..

[61] Pedersen O.F. (1997). The Peak Flow Working Group: Physiological determinants of peak expiratory flow. Eur. Respir. J..

[62] Willette R.N., Doorley B.M., Sapru H.N. (1987). Activation of cholinergic mechanisms in the medulla oblongata reverse intravenous opioid-induced respiratory depression. J. Pharmacol. Exp. Ther..

[63] Young A.P., Gruber R.B., Discala J.F., May W.J., McLaughlin D., Palmer L.A., Lewis S.J. (2013). Co-activation of μ- and δ-opioid receptors elicits tolerance to morphine-induced ventilatory depression via generation of peroxynitrite. Respir. Physiol. Neurobiol..

[64] Cragg P.A., Drysdale D.B. (1983). Interaction of hypoxia and hypercapnia on ventilation, tidal volume and respiratory frequency in the anaesthetized rat. J. Physiol..

[65] Wu M., Haxhiu M.A., Johnson S.M. (2005). Hypercapnic and hypoxic responses require intact neural transmission from the pre-Bötzinger complex. Respir. Physiol. Neurobiol..

[66] Blain G.M., Smith C.A., Henderson K.S., Dempsey J.A. (2010). Peripheral chemoreceptors determine the respiratory sensitivity of central chemoreceptors to CO_2_. J. Physiol..

[67] Smith C.A., Blain G.M., Henderson K.S., Dempsey J.A. (2015). Peripheral chemoreceptors determine the respiratory sensitivity of central chemoreceptors to CO_2_: Role of carotid body CO_2_. J. Physiol..

[68] Smith C.A., Forster H.V., Blain G.M., Dempsey J.A. (2010). An interdependent model of central/peripheral chemoreception: Evidence and implications for ventilatory control. Respir. Physiol. Neurobiol..

[69] Lydic R., Baghdoyan H.A., Wertz R., White D.P. (1991). Cholinergic reticular mechanisms influence state-dependent ventilatory response to hypercapnia. Am. J. Physiol..

[70] Trouth C.O., Millis R.M., Bernard D.G., Pan Y., Whittaker J.A., Archer P.W. (1993). Cholinergic-opioid interactions at brainstem respiratory chemosensitive areas in cats. Neurotoxicology.

[71] Getsy P.M., Coffee G.A., Bates J.N., Baby S.M., Seckler J.M., Palmer L.A., Lewis S.J. (2025). Functional evidence that S-nitroso-L-cysteine may be a candidate carotid body neurotransmitter. Neuropharmacology.

[72] Zarrindast M.R., Samadi P., Haeri-Rohani A., Moazami N., Shafizadeh M. (2002). Nicotine potentiation of morphine-induced catalepsy in mice. Pharmacol. Biochem. Behav..

[73] Zarrindast M.R., Fattahi Z., Rostami P., Rezayof A. (2005). Role of the cholinergic system in the rat basolateral amygdala on morphine-induced conditioned place preference. Pharmacol. Biochem. Behav..

[74] Rezayof A., Zatali H., Haeri-Rohani A., Zarrindast M.R. (2006). Dorsal hippocampal muscarinic and nicotinic receptors are involved in mediating morphine reward. Behav. Brain Res..

[75] Rezayof A., Nazari-Serenjeh F., Zarrindast M.R., Sepehri H., Delphi L. (2007). Morphine-induced place preference: Involvement of cholinergic receptors of the ventral tegmental area. Eur. J. Pharmacol..

[76] Horita A., Carino M.A., Chinn C. (1988). Codeine produces a cholinergically mediated analeptic effect in rats and rabbits. Pharmacol. Biochem. Behav..

[77] Horita A., Carino M.A., Chinn C. (1999). Fentanyl produces cholinergically-mediated analeptic and EEG arousal effects in rats. Neuropharmacology.

[78] Varagic V.M., Prostran M.S., Stepanovic S., Savic J., Vujnov S. (1991). Transmitter interactions in the central cholinergic control of blood pressure regulation. Drug Metabol. Drug Interact..

[79] Liu D., Zhou Y., Peng Y., Su P., Li Z., Xu Q., Tu Y., Tian X., Yang H., Wu Z. (2018). Endoplasmic Reticulum Stress in Spinal Cord Contributes to the Development of Morphine Tolerance. Front. Mol. Neurosci..

[80] Huang M., Luo L., Wang W., Xu H., Chen M., Ma X., Xu T. (2025). Targeting Excitatory Glutamate Receptors for Morphine Tolerance: A Narrative Review. CNS Neurosci. Ther..

[81] Casselini C.M., Parson H.K., Frizzi K.E., Marquez A., Smith D.R., Guernsey L., Nemmani R., Tayarani A., Jolivalt C.G., Weaver J. (2024). A muscarinic receptor antagonist reverses multiple indices of diabetic peripheral neuropathy: Preclinical and clinical studies using oxybutynin. Acta Neuropathol..

[82] Luo L., Zhang G., Mao L., Wang P., Xi C., Shi G., Leavenworth J.W. (2020). Group II muscarinic acetylcholine receptors attenuate hepatic injury via Nrf2/ARE pathway. Toxicol. Appl. Pharmacol..

[83] Stephenson R., Gucciardi E.J. (2002). Theoretical and practical considerations in the application of whole body plethysmography to sleep research. Eur. J. Appl. Physiol..

[84] Zysman-Colman Z., Lands L.C. (2016). Whole Body Plethysmography: Practical Considerations. Paediatr. Respir. Rev..

