# Supplementary material for: Role of Muscarinic Receptor Signaling Processes in Specific Aspects of Morphine-Induced Respiratory Depression in Rats
Source: Pharmaceuticals (Basel). 2026 Jul 17;19(7):1101. doi: 10.3390/ph19071101 (PMC13414587; doi:10.3390/ph19071101)
Supplement: Supplementary file 1 [file pharmaceuticals-19-01101-s001.zip › pharmaceuticals-4327870-supplementary.pdf]

Supplemental File

Role of Muscarinic Receptor Signaling Processes in Specific Aspects of Morphine-Induced Respiratory Depression in Rats

Paulina M. Getsy [1], Walter J. May [2], Santhosh M. Baby [3], Gregory A. Coffee [1], Yunguang Qiu [4,5,6], Feixiong Cheng [4,5,6], James N. Bates [7] and Stephen J. Lewis [1,8,*]

[1] Department of Pediatrics, Division of Pulmonology, Allergy and Immunology, School of Medicine, Biomedical Research Building, Case Western Reserve University, Cleveland, OH 44106, USA; pxg55@case.edu (P.M.G.); gac43@case.edu (G.A.C.)

[2] Department of Pediatrics, University of Virginia, Charlottesville, VA 22903, USA; walterjmay@gmail.com

[3] Galleon Pharmaceuticals, Inc., 213 Witmer Road, Horsham, PA 19044, USA; babysanthosh@gmail.com

[4] Genomic Medicine Institute, Lerner Research Institute, Cleveland Clinic, Cleveland, OH 44195, USA; qiuy3@ccf.org (Y.Q.); chengf@ccf.org (F.C.)

[5] Department of Molecular Medicine, Cleveland Clinic Lerner College of Medicine, Case Western Reserve University, Cleveland, OH 44195, USA

[6] Case Comprehensive Cancer Center, Case Western Reserve University School of Medicine, Cleveland, OH 44106, USA

[7] Department of Anesthesiology, University of Iowa Hospitals and Clinics, Iowa, IA 52242, USA; jnbates25@gmail.com

[8] Department of Pharmacology, Case Western Reserve University, Cleveland, OH 44106, USA

* Correspondence: sjl78@case.edu; Tel.: +1-843-422-7639

Supplemental Table S1

Updated list of the different classes of drugs that provide potential mechanisms of action in reversing opioid-induced respiratory depression (OIRD) and show promise in providing effective therapeutics for OIRD

**K^+-channel blockers**
Sia, R.L., Zandstra, D.F., 1981. 4-Aminopyridine reversal of fentanyl-induced respiratory depression in normocapnic and hypercapnic patients. Br. J. Anaesth. 53, 373-379. https://doi: 10.1093/bja/53.4.373<br>Roozekrans, M., van der Schrier, R., Okkerse, P., Hay, J., McLeod, J.F., Dahan, A. (2014). Two studies on reversal of opioid-induced respiratory depression by BK-channel blocker GAL021 in human volunteers. Anesthesiology 121, 459-468. https://doi: 10.1097/ALN.0000000000000367<br>Golder, F.J., Dax, S., Baby, S.M., Gruber, R., Hoshi, T., Ideo, C., Kennedy, A., Peng, S., Puskovic, V., Ritchie, D., Woodward, R., Wardle, R.L., Van Scott, M.R., Mannion, J.C., MacIntyre, D.E., 2015. Identification and Characterization of GAL-021 as a Novel Breathing Control Modulator. Anesthesiology 123, 1093-1104. https://doi: 10.1097/ALN.0000000000000844.<br>Roozekrans, M., Olofsen, E., van der Schrier, R., van Gerven, J., Peng, S., McLeod J., Dahan, A., 2015. Reversal of opioid-induced respiratory depression by BK-channel blocker GAL021: A pharmacokinetic-pharmacodynamic modeling study in healthy volunteers. Clin. Pharmacol. Ther. 97, 641-649. https://doi: 10.1002/cpt.99<br>Wei, A.D., Ramirez, J.M., 2019. Presynaptic Mechanisms and KCNQ Potassium Channels Modulate Opioid Depression of Respiratory Drive. Front. Physiol. 10, 1407. https://doi: 10.3389/fphys.2019.01407
**Acetylcholinesterase inhibition**
Elmalem, E., Chorev, M., Weinstock, M., 1991. Antagonism of morphine-induced respiratory depression by novel anticholinesterase agents. Neuropharmacology 30, 1059-1064. https://doi: 10.1016/0028-3908(91)90134-w<br>Tsujita, M., Sakuraba, S., Kuribayashi, J., Hosokawa, Y., Hatori, E., Okada, Y., Kashiwagi, M., Takeda, J., Kuwana, S., 2007. Antagonism of morphine-induced central respiratory depression by donepezil in the anesthetized rabbit. Biol. Res. 40, 339-346.<br>Sakuraba, S., Tsujita, M., Arisaka, H., Takeda, J., Yoshida, K., Kuwana, S., 2009. Donepezil reverses buprenorphine-induced central respiratory depression in anesthetized rabbits. Biol. Res. 42, 469-475.
**α_2-adrenoceptor antagonism**
Vonhof, S., Sirén, A.L., 1991. Reversal of µ-opioid-mediated respiratory depression by α_2-adrenoceptor antagonism. Life Sci. 49, 111-119. https://doi: 10.1016/0024-3205(91)90024-6

Phosphodiesterase inhibition

Kasaba, T., Takeshita, M., Takasaki, M., 1997. The effects of caffeine on the respiratory depression by morphine. Masui 46, 1570-1574.

Kimura, S., Ohi, Y., Haji, A., 2015. Blockade of phosphodiesterase 4 reverses morphine-induced ventilatory disturbance without loss of analgesia. Life Sci. 127, 32-38. https://doi: 10.1016/j.lfs.2015.02.006

Adenylate cyclase (cAMP producing) activator forskolin

Ballanyi, K., Lalley, P.M, Hoch, B., Richter, D.W., 1997. cAMP-dependent reversal of opioid- and prostaglandin-mediated depression of the isolated respiratory network in newborn rats. J. Physiol. 504, 127-134. https://doi: 10.1111/j.1469-7793.1997.127bf.x

Dopamine DA1 receptor agonists

Ballanyi, K., Lalley, P.M, Hoch, B., Richter, D.W., 1997. cAMP-dependent reversal of opioid- and prostaglandin-mediated depression of the isolated respiratory network in newborn rats. J. Physiol. 504, 127-134. https://doi: 10.1111/j.1469-7793.1997.127bf.x

Lalley, P.M., 2004. Dopamine1 receptor agonists reverse opioid respiratory network depression, increase CO_2 reactivity. Respir. Physiol. Neurobiol. 139, 247-262. https://doi: 10.1016/j.resp.2003.10.007

Lalley, P.M., 2005. D1-dopamine receptor agonists prevent and reverse opiate depression of breathing but not antinociception in the cat. Am. J. Physiol. Regul. Integr. Comp. Physiol. 289, R45-R51. https://doi: 10.1152/ajpregu.00868.2004

5-HT receptor agonists

Sahibzada, N., Ferreira, M., Wasserman, A.M., Taveira-DaSilva, A.M., and Gillis, RA., 2000. Reversal of morphine-induced apnea in the anesthetized rat by drugs that activate 5-hydroxytryptamine$_{1A}$ receptors. J. Pharmacol. Exp. Ther. 292, 704-713.

Manzke, T., Guenther, U., Ponimaskin, E.G., Haller, M., Dutschmann M., Schwarzacher, S., Richter, D.W., 2003. 5-HT4a receptors avert opioid-induced breathing depression without loss of analgesia. Science 301, 226-229. https://doi: 10.1126/science.1084674

Meyer, L.C., Fuller, A., Mitchell, D., 2006. Zacopride and 8-OH-DPAT reverse opioid-induced respiratory depression and hypoxia but not catatonic immobilization in goats. Am. J. Physiol Regul. Integr. Comp. Physiol. 290, R405-R413. https://doi: 10.1152/ajpregu.00440.2005

Dutschmann, M., Waki, H., Manzke, T., Simms, A.E., Pickering, A.E., Richter, D.W., Paton, J.F., 2009. The potency of different serotonergic agonists in counteracting opioid evoked cardiorespiratory disturbances. Philos. Trans. R. Soc. Lond. B. Biol. Sci. 364, 2611-2623. https://doi: 10.1098/rstb.2009.0076

Guenther, U., Manzke, T., Wrigge, H., Dutschmann, M., Zinserling, J., Putensen, C., Hoeft, A., 2009. The counteraction of opioid-induced ventilatory depression by the serotonin 1A-agonist 8-OH-DPAT does not antagonize antinociception in rats in situ and in vivo. Anesth. Analg. 108, 1169-1176. https://doi: 10.1213/ane.0b013e318198f828

Manzke, T., Niebert, M., Koch, U.R., Caley, A., Vogelgesang, S., Bischoff, A.M., Hülsmann, S., Ponimaskin, E., Müller, U., Smart, T.G., Harvey, R.J., Richter, D.W., 2011. Serotonin receptor 1A-modulated dephosphorylation of glycine receptor alpha3: a new molecular mechanism of breathing control for compensation of opioid-induced respiratory depression without loss of analgesia. Schmerz 25, 272-281. https://doi: 10.1007/s00482-011-1044-1<br>Guenther, U., Theuerkauf, N.U., Huse, D., Boettcher, M.F., Wensing, G., Putensen, C., Hoeft, A. (2012). Selective 5-HT_{1A}-R-agonist repinotan prevents remifentanil-induced ventilatory depression and prolongs antinociception. Anesthesiology 116, 56-64. https://doi: 10.1097/ALN.0b013e31823d08fa<br>Ren, J., Ding, X., Greer, J.J., 2015. 5-HT_{1A} receptor agonist Befiradol reduces fentanyl-induced respiratory depression, analgesia, and sedation in rats. Anesthesiology 122, 424-434. https://doi: 10.1097/ALN.0000000000000490
**Glycyl-glutamine**
Owen, M.D., Unal, C.B., Callahan, M.F., Trivedi, K., York, C., Millington, W.R., 2000. Glycyl-glutamine inhibits the respiratory depression, but not the antinociception, produced by morphine. Am. J. Physiol. 279, R1944-R1948. https://doi: 10.1152/ajpregu.2000.279.5.R1944
**Ampakines**
Ren, J., Poon, B.Y, Tang Y., Funk G.D., Greer, J.J., 2006. Ampakines alleviate respiratory depression in rats. Am. J. Respir. Crit. Care. Med. 174, 1384-1391. https://doi: 10.1164/rccm.200606-778OC<br>Greer, J.J., Ren, J., 2009. Ampakine therapy to counter fentanyl-induced respiratory depression. Respir. Physiol. Neurobiol. 168, 153-157. https://doi: 10.1016/j.resp.2009.02.011.<br>Ren J., Ding, X., Funk, G.D., Greer, J.J., 2000. Ampakine CX717 protects against fentanyl-induced respiratory depression and lethal apnea in rats. Anesthesiology 110, 1364-1370. https://doi: 10.1097/ALN.0b013e31819faa2a<br>Lorier, A.R., Funk, G.D., Greer, J.J., 2010. Opiate-induced suppression of rat hypoglossal motoneuron activity and its reversal by ampakine therapy. PLoS One 5, e8766. https://doi: 10.1371/journal.pone.0008766<br>Oertel, B.G., Felden, L., Tran, P.V., Bradshaw, M.H., Angst, M.S., Schmidt, H., Johnson, S., Greer, J.J., Geisslinger, G., Varney, M.A., Lötsch, J., 2010. Selective antagonism of opioid-induced ventilatory depression by an ampakine molecule in humans without loss of opioid analgesia. Clin. Pharmacol. Ther. 87, 204-211. https://doi: 10.1038/clpt.2009.194<br>Cavalla, D., Chianelli, F., Korsak, A., Hosford, P.S., Gourine, A.V., Marina, N., 2015. Tianeptine prevents respiratory depression without affecting analgesic effect of opiates in conscious rats. Eur. J. Pharmacol. 761, 268-272. https://doi: 10.1016/j.ejphar.2015.05.067.<br>Haw, A.J., Meyer, L.C., Greer, J.J., Fuller, A., 2016. Ampakine CX1942 attenuates opioid-induced respiratory depression and corrects the hypoxaemic effects of etorphine in immobilized goats (Capra hircus). Vet. Anaesth. Analg. 43, 528-538. https://doi: 10.1111/vaa.12358

Dai, W., Xiao, D., Gao, X., Zhou, X.B., Fang, T.Y., Yong, Z., Su, R.B., 2017. A brain-targeted ampakine compound protects against opioid-induced respiratory depression. Eur. J. Pharmacol. 809, 122-129. https://doi: 10.1016/j.ejphar.2017.05.025<br>Sun, Y., Liu, K., Martinez, E., Dale, J., Huang, D., Wang, J. (2017). AMPAkines and morphine provide complementary analgesia. Behav. Brain Res. 334, 1-5. https://doi: 10.1016/j.bbr.2017.07.020<br>Dai, W., Gao, X., Xiao, D., Li, Y.L., Zhou, X.B., Yong, Z., Su, R.B., 2019. The Impact and Mechanism of a Novel Allosteric AMPA Receptor Modulator LCX001 on Protection Against Respiratory Depression in Rodents. Front. Pharmacol. 10, 105. https://doi: 10.3389/fphar.2019.00105<br>Xiao, D., Xie, F., Xu, X., Zhou, X., 2020. The impact and mechanism of ampakine CX1739 on protection against respiratory depression in rats. Future Med. Chem. 12, 2093-2104. https://doi: 10.4155/fmc-2020-0256
**Microglial inhibitor**
Hutchinson, M.R., Northcutt, A.L., Chao, L.W., Kearney, J.J., Zhang, Y., Berkelhammer, D.L., Loram, L.C., Rozeske, R.R., Bland, S.T., Maier, S.F., Gleeson, T.T., Watkins, L.R., 2008. Minocycline suppresses morphine-induced respiratory depression, suppresses morphine-induced reward, and enhances systemic morphine-induced analgesia. Brain Behav. Immun. 22, 1248-1256. https://doi: 10.1016/j.bbi.2008.07.008
**NMDA receptor antagonist**
Jonkman, K., van Rijnsoever, E., Olofsen, E., Aarts, L., Sarton, E., van Velzen, M., Niesters, M., Dahan, A., 2018. Esketamine counters opioid-induced respiratory depression. Br. J. Anaesth. 120, 1117-1127. https://doi: 10.1016/j.bja.2018.02.021
**Inhibition of protein kinase A and GIRK channel**
Liang, X., Yong, Z., Su, R., 2018. Inhibition of protein kinase A and GIRK channel reverses fentanyl-induced respiratory depression. Neurosci. Lett. 677, 14-18. https://doi: 10.1016/j.neulet.2018.04.029
**Thyrotropin Releasing Hormone and Its Analog Taltirelin**
Boghosian, J.D., Luethy, A., Cotton, J.F., 2018. Intravenous and Intratracheal Thyrotropin Releasing Hormone and Its Analog Taltirelin Reverse Opioid-Induced Respiratory Depression in Isoflurane Anesthetized Rats. J. Pharmacol. Exp. Ther. 366, 105-112. https://doi: 10.1124/jpet.118.248377
**Nicotinic ion-channel receptor agonists**
Ren, J., Ding, X., Greer, J.J., 2019. Activating α4β2 Nicotinic Acetylcholine Receptors Alleviates Fentanyl-induced Respiratory Depression in Rats. Anesthesiology 130, 1017-1031. https://doi: 10.1097/ALN.0000000000002676<br>Ren, J., Ding, X., Greer, J.J., 2020. Countering Opioid-induced Respiratory Depression in Male Rats with Nicotinic Acetylcholine Receptor Partial Agonists Varenicline and ABT 594. Anesthesiology 132, 1197-1211. https://doi: 10.1097/ALN.0000000000003128

**Nitric Oxide Synthase**
Seckler, J.M., Grossfield, A., May, W.J., Getsy, P.M., Lewis, S.J. (2022). Nitrosyl factors play a vital role in the ventilatory depressant effects of fentanyl in unanesthetized rats. *Biomed. Pharmacother*. 146, 112571. https://doi: 10.1016/j.biopha.2021.112571
Getsy, P.M., May, W.J., Henderson, F. Jr., Seckler J.M., Grossfield, A., Baby, S.M., Lewis, S.J., 2025a. Nitrosyl factors play a vital role in the ventilatory depressant effects of fentanyl in freely moving guinea pigs. Biomed. Pharmacother. 183, 117847. https://doi: 10.1016/j.biopha.2025.117847
**S-nitrosothiols**
Getsy, P.M., Young, A.P., Bates, J.N., Baby, S.M., Seckler, J.M., Grossfield, A., Hsieh, Y.H., Lewis, T.H.J, Jenkins, M.W., Gaston, B., Lewis, S.J., 2022. S-nitroso-L-cysteine stereoselectively blunts the adverse effects of morphine on breathing and arterial blood gas chemistry while promoting analgesia. Biomed. Pharmacother. 153, 113436. https://doi: 10.1016/j.biopha.2022.113436
Getsy, P.M., Baby S.M., Gruber, R.B., Gaston, B., Lewis, T.H.J., Grossfield, A., Seckler, J.M., Hsieh, Y.H., Bates, J.N., Lewis, S.J., 2022. S-Nitroso-L-Cysteine Stereoselectively Blunts the Deleterious Effects of Fentanyl on Breathing While Augmenting Antinociception in Freely-Moving Rats. Front. Pharmacol. 13, 892307. https://doi: 10.3389/fphar.2022.892307
**Thiolesters**
Mendoza, J., Passafaro, R., Baby, S., Young, A.P., Bates, J.N., Gaston, B., Lewis, S.J., 2013. L-Cysteine ethyl ester reverses the deleterious effects of morphine on, arterial blood-gas chemistry in tracheotomized rats. Respir. Physiol. Neurobiol. 189, 136-143. https://doi: 10.1016/j.resp.2013.07.007
Gaston, B., Baby, S.M., May, W.J., Young, A.P., Grossfield, A., Bates, J.N., Seckler, J.M., Wilson, C.G., Lewis, S.J., 2021. D-Cystine di(m)ethyl ester reverses the deleterious effects of morphine on ventilation and arterial blood gas chemistry while promoting antinociception. Sci. Rep. 11, 10038. https://doi: 10.1038/s41598-021-89455-2
Jenkins, M.W., Khalid, F, Baby, S.M., May, W.J., Young, A.P., Bates, J.N., Cheng, F., Seckler, J.M., Lewis, S.J., 2021. Glutathione ethyl ester reverses the deleterious effects of fentanyl on ventilation and arterial blood-gas chemistry while prolonging fentanyl-induced analgesia. Sci. Rep. 11, 6985. https://doi: 10.1038/s41598-021-86458-x
Getsy P.M., Young A.P., Grossfield A., Seckler, J.M., Wilson, C.G., Gaston, B., Bates, J.N, Lewis, SJ., 2022c. D-cysteine ethyl ester and D-cystine dimethyl ester reverse the deleterious effects of morphine on arterial blood-gas chemistry and Alveolar-arterial gradient in anesthetized rats. Respir. Physiol. Neurobiol. 302, 103912. https://doi: 10.1016/j.resp.2022.103912
Getsy, P.M., Baby, S.M., May, W.J., Young, A.P., Gaston, B., Hodges, M.R., Forster, H.V., Bates, J.N., Wilson, C.G., Lewis, T.H.J, Hsieh Y.H., Lewis S.J., 2022d. D-Cysteine Ethyl Ester Reverses the Deleterious Effects of Morphine on Breathing and Arterial Blood-Gas Chemistry in Freely-Moving Rats. Front. Pharmacol. 13, 883329. https://doi: 10.3389/fphar.2022.883329

Getsy, P.M., Baby, S.M., May, W.J., Bates, J.N, Ellis, C.R., Feasel, MG., Wilson, C.G., Lewis, T.H.J., Gaston, B., Hsieh, Y.H., Lewis, S.J., 2022e. L-cysteine methyl ester overcomes the deleterious effects of morphine on ventilatory parameters and arterial blood-gas chemistry in unanesthetized rats. Front. Pharmacol. 13, 968378. https://doi: 10.3389/fphar.2022.968378<br>Getsy, P.M., Baby, S.M., May, W.J., Lewis, T.H.J., Bates, J.N., Hsieh, Y.H., Gaston, B., Lewis, S.J., 2022f. L-NAC reverses of the adverse effects of fentanyl infusion on ventilation and blood-gas chemistry. Biomed. Pharmacother. 153, 113277. https://doi: 10.1016/j.biopha.2022.113277<br>Lewis, T.H.J., May, W.J., Young, A.P., Bates, J.N., Baby, S.M., Getsy, P.M., Ryan, R.M., Hsieh, Y.H., Seckler, J.M., Lewis, S.J., 2022. The ventilatory depressant actions but not the antinociceptive effects of morphine are blunted in rats receiving intravenous infusion of L-cysteine ethyl ester. Biomed. Pharmacother. 156, 113939. https://doi: 10.1016/j.biopha.2022.113939
**Tropine and tropine ester**
Getsy, P.M., May, W.J., Young, A.P., Baby, S.M.., Coffee, GA., Bates, J.N., Hsieh, Y.H., Lewis, S.J., 2024a. Tropine exacerbates the ventilatory depressant actions of fentanyl in freely-moving rats. Front. Pharmacol. 15, 1405461. https://doi: 10.3389/fphar.2024.1405461<br>Getsy, P.M., May, W.J., Coffee, G.A., Baby, S.M., Hsieh, Y-H., Bates, J.N., Lewis, S.J., 2025. The ability of Ibutropin to blunt fentanyl-induced respiratory depression is independent of its activation of carotid body chemoafferents. J. Pharmacol. Exp. Ther. 392, 100060. https://doi.org/10.1016/j.jpet.2024.1000600022-3565<br>Getsy P.M., May W.J., Young, A.P., Baby, S.M., Lewis, T.H.J, McShine, A., Massien, S., Coffee, G.A., Bates, J.N., Hsieh, Y-H, Lewis, S.J., 2025. Isobutyric tropine ester (Ibutropin) overcomes fentanyl-induced respiratory depression in unanesthetized rats without compromising analgesia. Neuropharmacology, submitted.

Supplemental Table S2

Definition of ventilatory parameters

Parameter	Abbreviation	Units	Definition
**A. Directly recorded parameters**			
Frequency of breaths	Freq	breaths/min	Rate of breathing
Inspiratory Time	Ti	sec	Duration of inspiration
Expiratory Time	Te	sec	Duration of expiration
End Inspiratory Pause	EIP	msec	Pause between end of inspiration and start of expiration
End Expiratory Pause	EEP	msec	Pause between end of expiration and start of inspiration
Relaxation time	RT	sec	Decay of expiration to 36% maximum
Tidal Volume	TV	ml	Volume of inspired air per breath
Peak Inspiratory Flow	PIF	ml/sec	Maximum inspiratory flow
Peak Expiratory Flow	PEF	ml/sec	Maximum expiratory flow
Expiratory flow at 50%	EF_{50}	ml/sec	Expiratory flow at 50% expired TV
Rate of achieving PEF	Rpef	sec	How quickly PEF is achieved
Non-eupneic breathing index	NEBI	%	% of non-eupneic breaths per epoch
**B. Derived parameters**			
Minute Ventilation	MV = Freq x TV	ml/min	Total volume of air inspired per min
Ti/Te	Ti/Te	none	Inspiratory quotient
PIF/PEF	PIF/PEF	none	Flow balance
Expiratory Delay	Te-RT	No units	Difference in lengths of Te and RT
Inspiratory Drive	TV/Ti	ml/sec	Central urge to inhale
Expiratory Drive	TV/Te	ml/sec	Central urge to exhale
NEBI/Frequency	NEBI/Freq	%/(b/min)	Balanced rejection index

Supplemental Figure S1

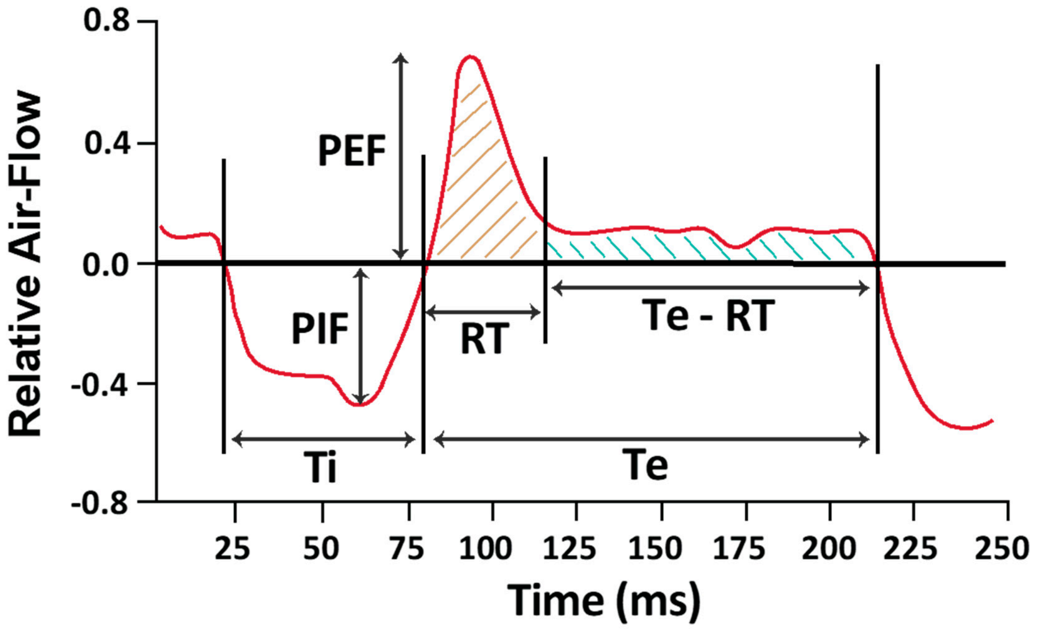

Supplemental Figure S1. Relationships between peak inspiratory flow (PIF), peak expiratory flow (PEF), relaxation time (RT) and expiratory time (Te).

Supplemental Table S3

Baseline ventilatory parameters

	Treatment Group	
**Parameter**	**Vehicle**	**Atropine**
Number	6	6
Age, days	83.2 ± 0.6	82.8 ± 0.5
Body Weight, gram	361 ± 2	359 ± 2
Frequency (Freq), breaths/min	92 ± 4	90 ± 2
Tidal Volume (TV), ml	2.71 ± 0.11	2.70 ± 0.10
Minute Ventilation, ml/min	247 ± 11	243 ± 10
Inspiratory Time (Ti), sec	0.243 ± 0.009	0.239 ± 0.008
Expiratory Time, sec	0.471 ± 0.027	0.490 ± 0.021
Inspiratory Time/Expiratory Time	0.53 ± 0.05	0.50 ± 0.04
End Inspiratory Pause, msec	8.1 ± 0.3	8.0 ± 0.2
End Expiratory Pause, msec	51.1 ± 2.4	51.7 ± 3.3
Peak Inspiratory Flow (PIF), ml/sec	14.7 ± 0.5	14.9 ± 0.2
Peak Expiratory Flow (PEF), ml/sec	11.9 ± 0.3	12.2 ± 0.4
PIF/PEF	1.23 ± 0.06	1.24 ± 0.05
Rate of achieving PEF (Rpef)	0.19± 0.01	0.19 ± 0.02
EF_{50}, ml/sec	0.44 ± 0.02	0.43 ± 0.03
Relaxation Time (RT), sec	0.31 ± 0.01	0.35 ± 0.02
Expiratory Delay (Te-RT)	0.16 ± 0.02	0.14 ± 0.02
Apneic Pause [Te/RT)-1]	0.52 ± 0.06	0.46 ± 0.04
Inspiratory Drive (TV/Ti), ml/sec	11.2 ± 0.6	11.4 ± 0.5
Expiratory Drive (TV/Te), ml/sec	5.9 ± 0.4	5.6 ± 0.3
Non-Eupneic Breathing Index (NEBI), % of epoch	4.4 ± 0.2	4.5 ± 0.1
NEBI/Freq, %/(breaths/min)	4.8 ± 0.2	5.0 ± 0.2

The data are presented as mean ± SEM. There were no between-group differences for any value (P > 0.05 for all comparisons).

Supplemental Table S4

Comparison of the effects of atropine in naïve and morphine-treated rats

	Treatment Group	
**Parameter**	**Naïve***	**Morphine-treated**
Frequency (Freq), breaths/min	↑↑↑↑	No effect
Tidal Volume (TV), ml	↓↓	No effect
Minute Ventilation, ml/min	↑↑	No effect
Inspiratory Time (Ti), sec	↓↓↓	No effect
Expiratory Time, sec	↓↓↓↓	No effect
Inspiratory Time/Expiratory Time	↑	No effect
End Inspiratory Pause, msec	↑	↓↓↓
End Expiratory Pause, msec	↓↓↓	No effect
Peak Inspiratory Flow (PIF), ml/sec	↑↑↑	No effect
Peak Expiratory Flow (PEF), ml/sec	↑↑↑	No effect
PIF/PEF	No effect	No effect
Rate of achieving PEF (Rpef)	↑↑↑↑	↓↓↓
EF_{50}, ml/sec	↑↑↑↑	↓
Relaxation Time (RT), sec	↓↓↓	No effect
Expiratory Delay (Te-RT)	↓↓↓	No effect
Apneic Pause [Te/RT)-1]	↓↓↓	No effect
Inspiratory Drive (TV/Ti), ml/sec	↑↑	No effect
Expiratory Drive (TV/Te), ml/sec	↑↑	No effect
Non-Eupneic Breathing Index (NEBI), % of epoch	↑↑↑↑	No effect
NEBI/Freq, %/(breaths/min)	↑↑	No effect

*Based on data from: Getsy PM, May WJ, Young AP, Baby SM, Coffee GA, Bates JN, Lewis SJ., 2025b. L-cysteine ethyl ester activates muscarinic receptor signaling processes to overcome morphine induced respiratory depression in freely-moving rats. Neuropharmacology, in press. ↑, stimulatory effect. ↓, inhibitory effect. The number of arrows is based on the %change in the raw data.
